# Exploring Germination to Unlock the Nutritional Potential of Sorghum (*Sorghum bicolor*)

**DOI:** 10.3390/molecules30173622

**Published:** 2025-09-04

**Authors:** Sara Margherita Borgonovi, Silvia Marzocchi, Federica Pasini, Alessandra Bordoni, Alberto Barbiroli, Alessandra Marti, Stefania Iametti, Mattia Di Nunzio

**Affiliations:** 1 Department of Food, Environmental and Nutritional Sciences (DeFENS), University of Milan, Via Celoria 2, 20133 Milan, Italy; sara.borgonovi@unimi.it (S.M.B.); alberto.barbiroli@unimi.it (A.B.); alessandra.marti@unimi.it (A.M.); stefania.iametti@unimi.it (S.I.); 2 Department of Agricultural and Food Sciences (DISTAL), University of Bologna, Piazza Goidanich 60, 47521 Cesena, Italy; silvia.marzocchi4@unibo.it (S.M.); federica.pasini5@unibo.it (F.P.); alessandra.bordoni@unibo.it (A.B.); 3 Interdepartmental Centre for Industrial Agri-Food Research (CIRI), University of Bologna, Piazza Goidanich 60, 47521 Cesena, Italy

**Keywords:** sprouting, starch, proteins, lipids, anti-nutritional factors, antioxidants

## Abstract

Thanks to its tolerance to drought, sorghum is a cereal crop that is extensively cultivated in the sub-Saharan region. Its good nutritional value makes it an interesting raw material for the food industry, although several anti-nutritional features pose a challenge to exploiting its full potential. In this study, we evaluated whether the process of germination may represent a way of improving the macro- and micro-molecular profile of sorghum, lowering the content of anti-nutritional factors, and promoting the synthesis of bioactive compounds. Germination for 48 and especially 72 h promoted the hydrolysis of starch and proteins, enhanced antioxidant activity, increased the content of polyphenols, mainly flavonols and flavanones, and promoted the conversion of γ- to α-isomers of tocopherols. At the same time, it significantly reduced the concentration of phytates and linoleic acid, enhancing pepsin activity and contributing to the inaugural examination of the impact of sprouted sorghum on digestive protease activity. These findings could help to promote the utilization of sprouted sorghum as a premium ingredient for food products, providing significant nutritional advantages.

## 1. Introduction

Future food security for humanity is under jeopardy due to the steady increase in the global population, the rarefaction of arable areas, and climate change [1]. To mitigate the risk of malnutrition resulting from food and nutritional insecurity, it is necessary to maximize the nutritional yield of crops. In this perspective, sorghum (*Sorghum bicolor*), a species of grass native to East Africa’s tropical and subtropical regions, is a good candidate. In fact, it has several agronomic advantages, including the ability to tolerate arid conditions, flexibility in planting time, resistance to pests and diseases, and low requirements for fertilizers or other agrochemicals [2].

In addition to its agronomic benefits, sorghum grain possesses a range of favorable nutritional attributes, including the absence of gluten for people with celiac disease and high content of minerals and bioactive phenolic compounds [3]. More intense sorghum utilization could encourage agricultural diversity, decrease environmental deterioration, and mitigate the effects of climate change on cereal production. In addition, sorghum has vast potential for exploitation and development into healthy and functional foods [4], and considering the growing consumer demand for healthy plant-based foods, it has garnered significant interest from both academia and the food industry over the past few decades.

As with many grains, sorghum is typically subjected to biotechnological pre-treatment, typically involving fermentation or sprouting (or germination), with the aim of enhancing flavor, structure, starch/protein digestibility, and the stability of baked goods [5,6]. Indeed, starch and proteins are linked in tightly packed complexes; therefore, an enzymatic pre-treatment step is often used in the preparation of a wide range of sorghum-based foods and beverages [7].

Biotechnological pre-treatment induces biochemical changes that reportedly contribute to the overall quality of sorghum-containing Western-style foods, such as pasta [2,8] or bread [9,10,11]. These treatments could also prove useful in overcoming the natural antinutritional factors present in sorghum, such as phytic acid and digestive protease inhibitors, which prevent the full exploitation of the technological and nutritional properties of the grain [12].

Several studies have investigated the physicochemical properties of macromolecules (proteins and starch) in sorghum flour upon fermentation or sprouting [13,14], but there is a paucity of studies aimed at a comprehensive characterization of the macro- and micro-biomolecular features of sprouted sorghum. In fact, studies in the literature have focused on just one or a few factors at a time, such as phytochemical content and phenolic profile [15], enzymatic activities [10], starch and proteins [16,17], and anti-nutritional factors such as phytic acid and tannins [18]. Moreover, the diverse experimental conditions employed during sprouting impede the execution of a consistent and exhaustive evaluation of the impact of this technology on biomolecular alterations. This hindrance complicates the interpretation of the events that occur during germination [19].

The present study was conducted with the objective of providing a wide, comprehensive overview of the alterations that occur in the macro- and micro-molecular profile, as well as the properties and content of bioactive compounds, in sprouted sorghum over a period of 48 or 72 h. Sprouting-related modifications in the content of anti-nutritional factors such as phytic acid and protease inhibitors, protein and carbohydrate hydrolysis, antioxidant compounds, and fatty acid profile were investigated. Moreover, to the best of our knowledge, this is the first study to have investigated the role of sprouted sorghum on the modulation of pepsin activity. The information provided herein will be fundamental for assessing the suitability of sprouted sorghum as a sustainable ingredient to be considered as a nutritious alternative to other major cereals for the preparation of baked goods.

## 2. Results and Discussion

### 2.1. Effects of Sprouting on Sorghum Starch

**Figure 1:**
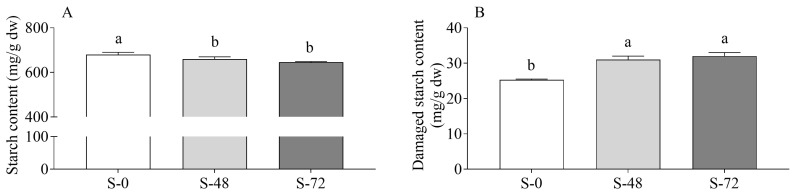
Starch (**A**) and damaged starch (**B**) content in unsprouted (S-0) and sprouted sorghum (48 h, S-48; 72 h, S-72). Data are expressed as mg/g dw and are the means ± SD of two different extractions and of triplicate spectrophotometric analysis. Statistical analysis was conducted by means of one-way ANOVA (always *p* < 0.05) with Tukey’s post hoc test (different letters indicate significant differences).

The total starch content of sorghum showed a decline after 48 h of sprouting, with no further evident decrease observed after 72 h (Figure 1A). This could be explained by the acceleration of the hydrolysis of endosperm starch into glucose, linked to the induction of α-amylase and α-glucosidase transcription in the aleurone by active gibberellin, thus providing energy for seed development [19,20]. Nevertheless, the trends observed in the sugar profile of sprouted grains are contingent on the species. For instance, rice, sorghum, and millet appear to accumulate more maltose than glucose, while buckwheat exhibits a greater propensity for glucose accumulation over maltose [21]. Despite the absence of a concomitant decline in total starch content, prior studies have documented a time-dependent escalation in α-amylase activity during the sprouting process [22]. In addition, the breakdown of starch during the sprouting process may also be influenced by the amylose/amylopectin ratio. In fact, sorghum starch has a high proportion of short amylopectin chains, which gives rise to a more porous granular structure, highly susceptible to enzymatic degradation [23]. This is consistent with the observed increase in damaged starch content (Figure 1B), which represents the fraction of starch that is physically accessible to amylases due to the presence of cavities in the outermost regions of starch granules [24].

The proposed impact of sprouting on the structure of sorghum starch granules was recently validated with scanning electron microscopy, which revealed fragmentation of the granules and the appearance of enlarged pores on their surfaces. These features are absent in the starch granules of unsprouted sorghum grains [25].

Our results collectively suggest that germination progressively reduced the proportion of resistant and slowly digestible starch, while concomitantly increasing the fractions of rapid digestible starch, as already observed in millet [26].

### 2.2. Effects of Sprouting on Sorghum Proteins

Prolamins and glutelins are sorghum main protein components, with prolamins dominating. The prolamin in sorghum is kafirin, a protein found primarily in the α- (Mr ≅ 21–23 kDa), β- (Mr ≅ 20 kDa), and γ- (Mr ≅ 47 kDa) isoforms. These subunits are organized in oligomers and polymers with a Mr of approximately 67–86 kDa [27].

**Figure 2:**
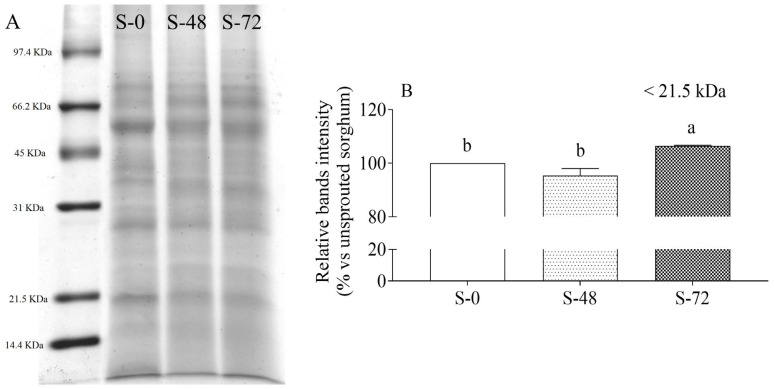
SDS-PAGE tracing (**A**) and relative bands intensity at ≤21.5 kDa protein fraction (**B**) in unsprouted (S-0) and sprouted sorghum (48 h, S-48; 72 h, S-72). Relative band intensity is expressed as the percentage of unsprouted sorghum (assigned as 100%) of two different extractions and SDS-PAGE analysis. Statistical analysis was conducted by means of one-way ANOVA (*p* < 0.05) with Tukey’s post hoc test (different letters indicate significant differences).

Non-reducing SDS-PAGE tracings (Figure 2A) showed multiple bands at an approximate Mr of 66, 55, 40, 28, and 20 kDa. The largest proteins were rather insensitive to sprouting-dependent proteolysis, except for the band detected at Mr ≅ 55 kDa, which showed a substantial decrease at 48 and 72 h of germination compared to the unsprouted condition (*p* < 0.05). Since β- and γ-kafirins are located on the outer part of the protein bodies, and they are the first to be hydrolyzed by proteases during sprouting [10], it can be assumed that the 55 kDa band originated from oligomers of β- and/or γ-isoforms. This is consistent with previous findings by Abdelbost et al. [28], who highlighted a temporal shift in kafirin breakdown, with the γ-kafirin peptides being released after 24–30 h of sprouting, well before α-kafirin (45 h). Considering the smaller amount of γ-kafirin compared to α-kafirin, this shift is noteworthy and fully consistent with the burial of α-kafirin within the protein bodies. After 72 h of sprouting, a significant increase in the relative intensity of the smaller protein fragment fraction with Mr ≤ 21.5 kDa was observed (Figure 2B), assuming hydrolysis of higher molecular weight proteins into smaller fragments.

**Figure 3:**
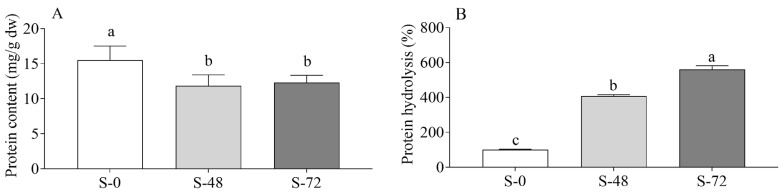
Protein content (**A**) and hydrolysis (**B**) in unsprouted (S-0) and sprouted sorghum (48 h, S-48; 72 h, S-72). Data are expressed as mg/g dw (**A**) and % with respect to unsprouted sorghum (assigned as 100%) (**B**). Data are the means ± SD of two different extractions and duplicate spectrophotometric analyses. Statistical analysis was conducted by means of one-way ANOVA (always: *p* < 0.05) with Tukey’s post hoc test (different letters indicate significant differences).

SDS-PAGE results were confirmed by means of the dye-binding assay, which detects large protein fragments (Mr > 3 kDa) [29], and the OPA assay, which also detects amino groups of small peptides/amino acids released during proteolysis [30]. By combining the results of the two assays, it was possible to monitor the decline in the content of large soluble proteins (Figure 3A), accompanied by the time-dependent increase in protein hydrolysis (Figure 3B).

Moreover, the process of protein hydrolysis during the germination of several types of plant food may be accompanied by the formation of peptides that possess biological activity, including antioxidants and antidiabetic properties [31].

These results confirmed that during plant germination, proteases are key protagonists. Indeed, the hydrolysis of proteins accumulated in seeds and cereal grains is fundamental for seed growth and plant development [32]. More than 90% of the endopeptidase activity in germinating seeds is accounted for by proteinases or endopeptidases, which are primarily sulfhydryl-dependent enzymes and play a significant role in the mobilization of seed storage proteins during germination [33]. In two wheat cultivars, sprouting has been demonstrated to enhance the hydrolytic activity in a time-dependent manner of two endogenous proteases at pH values of 4.4 and 7.5 over time [34]. As a consequence, it can be hypothesized that the increased degree of protein hydrolysis that occurs during the process of germination may be accompanied by a more efficient process of protein digestion. In this context, Bera et al. recently conducted a review of the ways in which germination can increase in vitro protein digestibility in both legumes and cereals, thereby demonstrating a strong relationship between proteolysis and digestion [35].

### 2.3. Effects of Sprouting on Sorghum Lipids and Lipid Oxidative Status

In agreement with Hassan et al. [36], the main fatty acids in sorghum were linoleic > oleic > palmitic acid, which, together, accounted for approximately 90% of total fatty acids. Although with significant species-dependent differences [37], sorghum sprouting caused a decrease in total fatty acid content, mainly related to the decrease in the essential fatty acid linoleic acid (C18:2 n-6), which was significant when comparing S-0 and S-72. Accordingly, the Σn-6/Σn-3 ratio decreased after germination (Table 1).

The decline in total fatty acid levels can be explained by the documented reactivation of triacylglycerol hydrolysis, fatty acid mobilization, glyoxalate cycle metabolism, and subsequent oxidation, which collectively allow for the conversion of stored fatty acids, facilitating the provision of energy during the sprouting process [38].

Although, from a nutritional point of view, the decrease in linoleic acid content could represent a negative aspect of sorghum germination, it is appropriate to consider the role of polyunsaturated fatty acids (PUFAs) on the stability and shelf life of the flour. During the processing of flour, fats can undergo oxidation, leading to rancidity and the development of off-flavors. Consequently, a decrease in total PUFA content can enhance the flour stability, extending the shelf life of the flour and products manufactured from it. This is particularly important considering that the transition from a quiescent seed to a metabolically active organism is associated with the generation of reactive species, the production of which has been reported during the germination period of cereals and legumes [39,40,41].

In this study, although the sprouting process resulted in a notable decrease in oxidation-sensitive fatty acids, the level of CD-containing lipids, which are sensitive indicators of lipid peroxidation [42], significantly increased after 72 h of sprouting (S-0: 100.00 ± 16.00 b; S-48: 118.45 ± 2.13 ab; S-72: 128.22 ± 1.17 a). Rancidity resulting from the oxidation of fats may affect the taste and aroma of the flour due to off-flavor development [43]; therefore, it is recommended to carry out sensory analyses with the utmost care when developing food formulations with sprouted sorghum.

### 2.4. Effects of Sprouting on Sorghum Antioxidants

In agreement with Niro et al. [44], γ- and α-tocopherols were the two most abundant tocols in quiescent and sprouted sorghum, respectively. The increase in α-isomers was associated with a decrease in γ-tocopherol, without any modification in total tocol content (Table 2).

Although the mechanisms by which sprouting modified the concentration and profile of vitamin E in sorghum remain unclear [25], the conversion of γ-tocopherol to α tocopherol can be primarily attributed to the elevated expression of the gamma-tocopherol methyltransferase gene during germination [45]. However, a similar effect has been observed in wheat and amaranth [46]. Considering that tocols other than α-form are not or poorly absorbed by humans [47], whatever the mechanism, the modification of the vitamin E profile represents an improvement of the nutritional quality of sorghum related to germination.

Tocols are among the most important lipid-soluble antioxidants, and several studies have demonstrated that an increase in the levels of these compounds may effectively prevent lipid oxidation [48]. Given the potential for genetic diversity and cultural techniques to influence the content of tocols in sorghum [49], the selection of optimal conditions may prove an effective strategy to mitigate lipid oxidation during the sprouting process by increasing the concentration of these lipophilic antioxidants.

Phenolic compounds are one of the main antioxidants in plants, and their composition and content in cereals vary depending on variety and agricultural and storage conditions [50]. In this study, 17 phenolic compounds—representative of eight classes—were identified and quantified in sorghum (Table 3 and Table 4). In unsprouted sorghum, the amount of free phenolic compounds was 28 times higher than that of the bound species, and the most representative free phenolic classes were flavonols > flavones > phenolic acids, which together accounted for about 96% of the total free phenolic content.

The process of germination resulted in a notable elevation in the concentration of phenolic compounds, both in the free and bound fractions. The main free phenolic classes were flavonols > flavones > flavanones (approximately 94% of total free phenolic compounds), while the bound phenolics were flavones > flavanones > flavonols.

Sprouting determined a time-dependent decrease in total free phenolic acids without affecting total free flavanonols, bound phenolic acids, bound flavan-3-ols, bound proanthocyanidins, bound flavanonols, and bound 3-deoxyanthocyanidins. On the contrary, total free and bound flavones and total bound phenol content increased after 72 h of sprouting. Total free flavan-3-ols, free and bound flavonols, free proanthocyanidins, free and bound flavanones, free flavanonols, free 3-deoxyanthocyanidins, and total free phenols content appeared at the highest concentration just after 48 h of sprouting.

A significant increase in polyphenols had already been observed after the germination of other cereals [51,52,53], and it could be related to the activation of phenylalanine ammonia lyase (PAL), a crucial enzyme in phenolic biosynthesis through the conversion of phenylalanine to trans-cinnamic acid and ammonia [54]. A positive correlation between PAL activity and the content of total polyphenols was evidenced in wheat [55]. Additionally, Dicko et al. [56] evaluated PAL activity in fifty varieties of sorghum, detecting it in only 50% of the ungerminated varieties and in all varieties after sprouting.

Sprouting resulted in a time-dependent increase in antioxidant capacity (S-0: 9.0 ± 0.24 a µmol Trolox eq/g dw; S-48: 14.98 ± 1.67 b µmol Trolox eq/g dw; S-72: 19.35 ± 0.61 c µmol Trolox eq/g dw), which did not correlate with the free phenolic content (Pearson r = 0.7811, r^2^ = 0.6102, *p* = 0.4293). The lack of association between TAC and free phenolic content could be related to the presence of other water-extractable bioactive components with radical scavenger capacity, such as ascorbic acid [57]. Although grains are not considered a source of ascorbic acid, some studies have suggested an increase in this antioxidant upon the sprouting of barley and wheat, particularly when exposed to light during germination [58]. In addition, when evaluating the scavenger capacity of polyphenols, in addition to their concentration, the relationship between their chemical structure and antioxidant activity must be carefully considered [59]. In particular, the chemical substituents in polyphenol appear to have a stronger influence on their antioxidant action than the backbone. In fact, Platzer et al. provided evidence that the Bors criteria were less accurate at predicting antioxidant activity than the quantity of hydroxyl groups present in the molecules [60].

### 2.5. Effects of Sprouting on Sorghum Anti-Nutritional Factors

In cereals and legumes, phytic acid is the primary phosphorus storage, which serves the biosynthetic needs of growing tissues during germination [61]. Phytic acid is generally considered an anti-nutritional factor as it binds to minerals, proteins, and starch, limiting their bioavailability [62]. In sorghum, phytate content significantly decreased after 48 h and 72 h of sprouting compared to the unsprouted seeds (S-0: 9.58 ± 0.01 a mg/g dw; S-48: 7.11 ± 0.38 b mg/g dw; S-72: 6.39 ± 0.30 b mg/g dw). The observed decreases in phytate concentrations can be attributed to the leakage of water-soluble phytate during soaking [63] and to the activation, de novo synthesis, and accumulation of phytase to produce free inorganic phosphorus, along with a chain of intermediate myo-inositol phosphates [64,65].

Given that cereals are a common source of protease inhibitors [66], the impact of sorghum extracts on the activity of pepsin—the primary protease in human gastric juice [67]—was assessed. The addition of unsprouted sorghum extract had no effect on enzyme activity, whereas the inclusion of sprouted sorghum resulted in a notable increase (Table 5).

It can be hypothesized that the observed modulation of pepsin activity may be at least partly due to the increased free polyphenol content during germination. In a recent study, Gomez-Urios et al. demonstrated that a polyphenol-rich extract from orange juice peel was capable of enhancing pepsin activity, but not trypsin and chymotrypsin [68]. Furthermore, Borgonovi et al. recently conducted a comparative study to estimate the modulatory effect of twenty-five bioactives on digestive protease activity using different substrates. The present investigation, employing an integrated in vitro and in silico approach, revealed that bioactive compounds may exhibit contradictory effects on proteolytic activity, contingent on the substrate/enzyme combination and the configuration of the bioactivities [69].

Although the ameliorative effect of sprouting on phytate content and trypsin inhibition in cereals and legumes has been deeply explored so far [58,63,70,71,72], to the best of our knowledge, this is the first investigation reporting the effect of sorghum sprouting on pepsin activity, and our findings corroborate recent reports on increased in vitro protein digestibility in sprouted wheat [73] and brown finger millet [74].

## 3. Materials and Methods

### 3.1. Materials

Unless otherwise stated, chemicals and solvents were of the highest analytical grade and were from Merck KGaA (Darmstadt, Germany). Dehulled commercial sorghum seeds were kindly provided by Molino Filippini s.r.l. (Teglio, Sondrio, Italy).

### 3.2. Sprouting Process

Seeds were sprouted in a lab-scale climate chamber (IPP110ecoplus, Memmert GmbH Co. KG, Schwabach, Germany). The seeds were soaked in water (seed–water ratio of 1:3 *w*/*w*) for 16 h at 27 °C. After removing excess water, the seeds were sprouted for 48 h (S-48) and 72 h (S-72) at 27 °C and 90% relative humidity [51].

The optimal sprouting times were determined in previous research, which indicated that times below 48 h may yield unsatisfactory outcomes [75], while times exceeding 72 h can lead to substantial deterioration in flour characteristics, dough quality, and bread-baking performance [76,77]. After sprouting, seeds were dried at 50 °C for 8 h in an oven (Self Cooking Center^®^, Rational International AG, Landsberg am Lech, Germany). Soaking, sprouting, and drying parameters are summarized in Supplementary Table S1.

Two batches for each sprouting time were produced, and the seeds were mixed. Unsprouted sorghum was used as a control (S-0). All samples were milled into flour (<0.5 mm) using a laboratory mill (IKA Universalmühle M20; IKA Laborteknic, Staufen, Germany). Dry weight (dw) was measured according to the AACC 08-01 method and was 0.87 g dw/g flour (S-0), 0.91 g dw/g flour (S-48), and 0.91 g dw/g flour (S-72).

### 3.3. Total Starch Content

The total starch content was measured according to the AACC 76-13.01 method using the Total Starch Assay Kit (Megazyme International Ltd., Bray, Ireland) following the manufacturer’s instructions [78]. The method is based on the quantification of glucose content using a glucose oxidase/peroxidase reagent, after starch hydrolysis with α-amylase and amyloglucosidase. All values obtained were normalized for g dry weight.

### 3.4. Damaged Starch Content

Damaged starch content was measured according to the AACC 76-31.01 using the Starch Damage Assay Kit (Megazyme International Ltd., Bray, Ireland) following the manufacturer’s instructions [79]. In this method, damaged starch granules are hydrated and then subjected to hydrolysis by fungal α-amylase to produce maltosaccharides and limit dextrins. Amyloglucosidase is then utilized for the purpose of converting dextrins into glucose. The spectrophotometric determination of glucose is achieved through the utilization of glucose oxidase/peroxidase reagent. All values obtained were normalized for g dry weight.

### 3.5. Sodium Dodecyl Sulfate-Polyacrylamide Gel Electrophoresis (SDS-PAGE) of Total Proteins

Sorghum flour was subjected to total protein extraction in duplicate by suspending 2 mg of the samples in 50 µL of 0.5 M TRIS-HCl buffer (pH 6.8) and 50 µL of denaturing buffer (0.125 M Tris-HCl, pH 6.8, 50% glycerol, 1.7% SDS, 0.01% bromophenol blue). Samples were heated at 95 °C for 5 min and centrifuged at 13,000 rpm for 10 min. SDS-PAGE was carried out using home-cast 12% polyacrylamide gels [80]. Gels were stained with Coomassie Blue R250, and a grayscale image of the polyacrylamide gels was obtained through a benchtop image scanner. Images of each lane were vertically divided into four portions, according to their molecular mass (Mr): total (Mr ≤ 97.4 kDa), high (97.4 kDa < Mr ≤ 45 kDa), medium (45 kDa < Mr ≤ 21.5 kDa), and low (Mr < 21.5 kDa). After background color correction, the grey intensity, integrated over the corresponding pixels in each portion, was evaluated through the image analyzer program Image Lab software version 6.1 (Bio-Rad Laboratories, Hercules, CA, USA).

### 3.6. Protein Content and Hydrolysis Degree

Sorghum flour was subjected to aqueous extraction in duplicate by suspending 0.5 g of the samples in 5 mL of 0.05 M sodium phosphate buffer (pH 7) containing 0.1 M NaCl. After 1 h of stirring at 25 °C, the suspension was centrifuged (2500× *g*, 30 min, 25 °C). The total amount and the hydrolysis degree of water-soluble proteins in the aqueous sorghum extract were determined spectrophotometrically with Coomassie Blue reagent [81] and o-phthaldialdehyde (OPA) [82] assays using bovine serum albumin and L-isoleucine as the standards, respectively. Protein content and hydrolysis degree are expressed as mg/g dry weight and as the percent of unsprouted flour (assigned as 100%), respectively.

### 3.7. Fatty Acid Content and Composition

Total lipids were extracted according to the method of Bligh & Dyer [83]. After methylation [84], the content and profile of fatty acids (as fatty acid methyl esters—FAMEs) were determined by means of gas chromatography (GC) (GC-2030AF; Shimadzu, Kyoto, Japan) using a capillary column (SP2340, L × I.D. 30 m × 0.25 mm, df 0.20 μm) with a programmed temperature gradient (50–250 °C, 10 °C/min) [85]. The GC peaks were identified based on their retention time using Supelco 37 Component FAME Mix. Quantitative evaluations were obtained using Lab Solution software (Shimadzu, Kyoto, Japan) using pentadecaenoic acid as the internal standard. Peroxidizability and unsaturation index were calculated as previously reported [86].

### 3.8. Lipid Peroxidation

Lipid peroxidation was assessed by quantifying conjugated dienes (CDs) as a marker of lipid peroxidation [87]. One hundred mg of sorghum flour was thoroughly mixed with 3.6 mL hexane/isopropanol solution (3:1 *v*/*v*) before adding 2.4 mL of a 7% (*w*/*v*) sodium sulphate anhydrous solution. After phase separation, the upper layer was collected and transferred to a test tube. The hexane/isopropanol layer was evaporated under nitrogen infusion, and lipids were redissolved in 5 mL isooctane. Absorbance was measured at 232 nm against a blank (isooctane) and expressed as the percent of unsprouted flour (assigned as 100%).

### 3.9. Phytic Acid Content

Phytic acid as total phosphorus was determined using the Phytic Acid/Total Phosphorus Kit (Megazyme International Ltd., Bray, Ireland) according to the manufacturer’s instructions [88]. The method is a dual-step reaction comprising phytase and alkaline phosphatase, with the objective of yielding 12-molybdophosphoric acid. Thereafter, the 12-molybdophosphoric acid is converted to molybdenum blue under acidic and reducing conditions and measured spectrophotometrically at a wavelength of 655 nm (Infinite 200, Tecan Life Sciences, Männedorf, Switzerland). All values obtained were normalized for g dry weight.

### 3.10. Pepsin Activity

Pepsin activity was determined based on the stop-point assay of hemoglobin degradation [89]. First, 400 μL of 2% (*w*/*v*) bovine blood hemoglobin (pH 2) was added to 20 μL of aqueous sorghum extract, and the reaction started with 100 μL of pepsin at 30 µg/mL. After 10, 20, and 30 min, the reaction was stopped by adding 1 mL of 20% (*w*/*v*) trichloroacetic acid (TCA). After centrifugation (12,000× *g* for 10 min), TCA-soluble peptides in the supernatant were detected at 280 nm (Infinite 200, Tecan Life Sciences, Männedorf, Switzerland). Pepsin activity was expressed as Unit, where one unit determines a ΔA280 of 0.001/min, and normalized for mg of enzyme.

### 3.11. Tocol Extraction and Determination via HPLC–FLD

Tocols were evaluated using an HPLC 1200 series apparatus equipped with a fluorimeter detector (FLD) (λ_ex_ = 290 nm, λ_em_ = 325 nm) according to the method of Di Nunzio et al. [90]. Of note, 50 mg of oil dissolved in 0.5 mL of hexane was homogenized with a vortex and filtered through a 0.20 μm nylon filter in vials. The separation of tocols was performed using an HILIC Poroshell 120 column (100 mm× 3 mm and 2.7 μm particle size; Agilent Technologies, Santa Clara, CA, USA), under isocratic conditions, with a n-hexane/ethyl acetate/acetic acid (97.3:1.8:0.9 *v*/*v*/*v*) mobile phase and a flow rate of 0.8 mL/min. The individual tocols were identified by means of co-elution with the respective standards, and the calibration curve of α-tocopherol standard solutions was used for their quantification. The values obtained were normalized for g dry weight.

### 3.12. Extraction and Determination of Free and Bound Phenolic Compounds

Free and bound phenolic compounds were extracted and analyzed as reported by Borgonovi et al. [51]. Of note, 2 g of sample was extracted in an ultrasonic bath at 40 °C with ethanol/water (4:1, *v*/*v*) for 10 min. The supernatants were collected, evaporated, and reconstituted with 2 mL of methanol/water (1:1, *v*/*v*). The extracts were stored at −18 °C until use. Residues of free phenolic extraction were digested overnight (20 h) with 200 mL of 2 M NaOH at room temperature under nitrogen to obtain the bound phenolic fraction. The hydrolyzed solution was acidified to pH 2 with hydrochloric acid in a cooling ice bath and extracted five times with 100 mL of ethyl acetate. The organic fractions were pooled, evaporated, and finally reconstituted in 2 mL of methanol/water (1:1, *v*/*v*).

Separation of free and bound phenolic compounds from sorgo powder was carried out using a C-18 column (Poroshell 120, SB-C18, 3.0 × 100 mm, 2.7 µm from Agilent Technologies, Palo Alto, CA, USA) and an Agilent HPLC 1290 series. MS/MS analysis (MRM mode) was performed on a 6420 Triple Quadrupole (Agilent Technologies, Santa Clara, CA, USA) using an electrospray ionization (ESI) interface in negative and positive ionization mode. Ferulic acid, catechin, and rutin were used as standards for the quantification of the different compounds depending on the phenolic class they belong to. Values obtained were normalized for g dry weight.

### 3.13. Total Antioxidant Capacity (TAC)

TAC was measured based on the ability of the antioxidant molecules in the sample to reduce the radical cation of 2,2′-azinobis-(3-ethylbenzothiazoline-6-sulfonic acid) (ABTS•+) [68]. TAC was determined on 0.01 mL of the aqueous sorghum extract by evaluating spectrophotometrically the discoloration of ABTS•+ at 734 nm (Infinite 200, Tecan Life Sciences, Männedorf, Switzerland). The values obtained were compared to the concentration–response curve of a standard solution of 6-hydroxy-2,5,7,8-tetramethylchroman-2-carboxylic acid (Trolox). The values obtained were normalized for g dry weight.

### 3.14. Statistical Analysis

Statistical analysis was conducted by means of the one-way analysis of variance (ANOVA) followed by Tukey’s post hoc test using GraphPad Prism 10 (Boston, MA, USA) and considering at least *p* < 0.05 as significant.

## 4. Conclusions

Sprouting resulted in significant compositional changes in sorghum seeds. The substantially increased release of free peptides/amino acids and phenolic compounds with antioxidant activity and the reduction in antinutritional factors make sprouting appear to be a good procedure to enhance the nutritional qualities of sorghum and produce a high-quality functional ingredient. It is particularly noteworthy that the increase in pepsin activity observed with germinated sorghum extracts may represent a new field of study regarding the creation of foods with higher protein digestibility. Under the experimental conditions, although some ameliorative modifications had already reached their maximum after 48 h, it was observed that 72 h was the optimal sprouting time for increasing protein hydrolysis, bound polyphenol content, and antioxidant capacity, as well as reducing the total fatty acid content. The findings presented herein may prove useful in developing sustainable methods for implementing optimal nutritional characteristics using bioprocesses, with the aim of satisfying changing consumer needs and the impact of climate change on the availability of novel plant-based raw materials. Further studies, including the evaluation of the digestibility of sprouted material, the identification of bioactive species in proteolytic fragments (i.e., bioactive peptides), and the effect on the gut microbiota, are needed to boost the industrial exploitation of sorghum and traditional sorghum-based foods. Such studies would be particularly beneficial for low-income countries where sorghum is a staple crop, offering significant economic and nutritional security advantages.

## Figures and Tables

**Table 1:** Fatty acid (as fatty acid methyl esters, FAMEs) in unsprouted (S-0) and sprouted sorghum (48 h, S-48; 72 h, S-72). Fatty acid content is expressed as mg FAME/g dw. Data are the means ± SD of two different extractions and duplicate gas chromatographic analyses. Statistical analysis was conducted by means of one-way ANOVA (C18:2 n-6, ΣPUFA, Σn-6/Σn-3, total FAME: *p* < 0.05) with Tukey’s post hoc test (different letters indicate significant differences). n.d.: not detected; SFAs: saturated fatty acids; MUFAs: monounsaturated fatty acids; PUFAs: polyunsaturated fatty acids; UI: unsaturation index; PI: peroxidizability index.

FAME	S-0	S-48	S-72
C14:0	n.d. a	0.08 ± 0.04 a	n.d. a
C16:0	3.95 ± 0.06 a	3.91 ± 0.47 a	3.23 ± 0.01 a
C16:1 n-7	0.06 ± 0.09 a	0.12 ± 0.18 a	0.05 ± 0.07 a
C18:0	n.d. a	0.35 ± 0.14 a	0.66 ± 0.29 a
C18:1 n-9	8.04 ± 0.05 a	7.73 ± 1.30 a	5.88 ± 0.04 a
C18:2 n-6	12.13 ± 1.01 a	10.75 ± 0.27 ab	9.29 ± 0.12 b
C18:3 n-3	0.36 ± 0.05 a	0.46 ± 0.00 a	0.43 ± 0.02 a
ΣSFAs	3.95 ± 0.06 a	4.34 ± 0.37 a	3.89 ± 0.28 a
ΣMUFAs	8.11 ± 0.14 a	7.85 ± 1.48 a	5.93 ± 0.03 a
ΣPUFAs	12.49 ± 1.06 a	11.20 ± 0.27 ab	9.72 ± 0.10 b
Σn-6/Σn-3	33.64 ± 1.75 a	23.49 ± 0.47 b	21.71 ± 1.18 b
Total	24.55 ± 0.98 a	23.40 ± 1.58 ab	19.53 ± 0.35 b
UI	136.21 ± 2.83 a	131.45 ± 4.85 a	132.04 ± 1.59 a
PI	60.57 ± 1.96 a	58.35 ± 3.51 a	59.53 ± 0.68 a

**Table 2:** Tocol content in unsprouted (S-0) and sprouted sorghum (48 h, S-48; 72 h, S-72). Data are expressed as µg/g dw and are the means ± SD of two extractions and duplicate chromatographic runs. Statistical analysis was conducted by means of one-way ANOVA (α-tocopherol, α-tocotrienol, γ-tocopherol, and δ-tocopherol: *p* < 0.05) with Tukey’s post hoc test (different letters indicate significant differences).

Tocols	S-0	S-48	S-72
α-tocopherol	3.47 ± 0.00 b	11.53 ± 0.43 a	10.44 ± 0.18 a
α-tocotrienol	1.98 ± 0.10 b	3.56 ± 0.21 a	3.20 ± 0.18 a
γ-tocopherol	14.77 ± 0.24 a	5.05 ± 0.64 b	3.03 ± 0.00 c
δ-tocopherol	1.78 ± 0.27 b	2.63 ± 0.17 a	2.54 ± 0.00 ab
Total	22.00 ± 0.61 a	22.77 ± 1.45 a	19.21 ± 0.36 a

**Table 3:** Free phenol content in unsprouted (S-0) and sprouted sorghum (48 h, S-48; 72 h, S-72). Data are expressed µg/g dw and are the means ± SD of two extractions and duplicate chromatographic analyses. Statistical analysis was conducted by means of one-way ANOVA with Tukey’s post hoc test (different letters indicate significant differences). Q.T.: quantification transition.

Compounds	[M/H]^−^	MS Fragments	Q. T.	Free Phenolic Compounds			ANOVA
				S-0	S-48	S-72	
Phenolic acids							
1-3-O-dicaffeoylglycerol	415	161, 253, 135	415 → 135	270.10 ± 8.46 a	72.41 ± 0.63 b	23.83 ± 0.64 c	*p* < 0.05
Caffeic acid	179	135	179 → 135	35.92 ± 1.58 a	16.02 ± 0.05 b	3.90 ± 0.35 c	*p* < 0.05
Total phenolic acid				306.02 ± 10.04 a	88.43 ± 0.68 b	27.73 ± 0.99 c	*p* < 0.05
Flavan-3-ols							
Catechin	289	123, 109, 203	289 → 123	n.d. b	33.99 ± 1.70 a	n.d. b	*p* < 0.05
Total flavan-3-ol				n.d. b	33.99 ± 1.70 a	n.d. b	*p* < 0.05
Flavonols							
Taxifolin	303	125, 175, 217	303 → 125	1327.69 ± 45.66 c	2233.50 ± 9.50 a	1803.68 ± 42.04 b	*p* < 0.05
Total flavonol				1327.69 ± 45.66 c	2233.50 ± 9.50 a	1803.68 ± 42.04 b	*p* < 0.05
Flavones							
Apigenin	269	117, 149, 227	269 → 117	23.86 ± 2.27 b	21.72 ± 0.59 b	29.79 ± 0.30 a	*p* < 0.05
Hispidulin isomer 1	299	284, 256, 136	299 → 284	58.23 ± 8.18 c	122.05 ± 5.37 b	179.52 ± 0.67 a	*p* < 0.05
Hispidulin isomer 2	299	284, 256, 136	299 → 284	188.27 ± 13.19 b	150.60 ± 7.90 b	281.74 ± 23.64 a	*p* < 0.05
Luteolin	285	133, 151, 217	285 → 133	373.47 ± 18.47 a	348.81 ± 4.69 a	435.66 ± 33.45 a	*p* < 0.05
Total flavones				643.83 ± 42.11 b	643.18 ± 18.55 b	926.71 ± 58.06 a	*p* < 0.05
Proanthocyanidins							
Procyanidin dimer	577	289, 407, 425	577 → 289	n.d. c	36.08 ± 3.54 a	26.96 ± 3.44 b	*p* < 0.05
Total proanthocyanidins				n.d. c	36.08 ± 3.54 a	26.96 ± 3.44 b	*p* < 0.05
Flavanones							
Eriodictyol	287	125, 151, 193	287 → 125	40.51 ± 3.29 c	219.26 ± 3.19 a	202.01 ± 2.77 b	*p* < 0.05
Naringenin	271	119, 107, 151	271 → 119	5.83 ± 0.49 b	2.02 ± 0.03 b	37.82 ± 2.09 a	*p* < 0.05
Kaempferol	285	199, 175, 217	285 → 199	4.48 ± 0.56 c	18.71 ± 0.14 a	10.41 ± 1.25 b	*p* < 0.05
Total flavanones				50.82 ± 4.34 b	239.99 ± 3.36 a	250.24 ± 6.11 a	*p* < 0.05
Flavanonols							
Dihydromyricetin 3-O-Rhamnoside	465	285, 275, 303	465 → 285	31.98 ± 1.31 a	34.30 ± 1.38 a	41.13 ± 5.13 a	*p* < 0.05
Total flavanonols				31.98 ± 1.31 a	34.30 ± 1.38 a	41.13 ± 5.13 a	*p* < 0.05
3-Deoxyanthocyanidins							
Luteolinidin	269	201, 227, 241	269 → 201	2.23 ± 0.16 c	4.96 ± 0.62 b	7.64 ± 0.54 a	*p* < 0.05
Apigeninidin	253	209, 117, 181	253 → 209	6.51 ± 0.59 a	5.04 ± 0.50 a	1.29 ± 0.10 b	*p* < 0.05
5-methoxy-luteolinidin	283	268, 196, 240	283 → 268	6.02 ± 0.10 a	7.08 ± 0.22 a	3.68 ± 0.50 b	*p* < 0.05
7-methoxy-apigeninidin	267	252, 224, 180	267 → 252	1.58 ± 0.31 c	3.10 ± 0.43 b	5.25 ± 0.30 a	*p* < 0.05
Total 3-deoxyanthocyanidins				16.34 ± 1.16 b	20.18 ± 1.77 a	17.86 ± 1.44 b	*p* < 0.05
Total phenol compounds				2376.68 ± 104.62 c	3329.65 ± 40.48 a	3104.31 ± 3117.21 b	*p* < 0.05

**Table 4:** Bound phenol content in unsprouted (S-0) and sprouted sorghum (48 h, S-48; 72 h, S-72). Data are expressed µg/g dw and are the means ± SD of two extractions and duplicate chromatographic analyses. Statistical analysis was conducted by means of one-way ANOVA with Tukey’s post hoc test (different letters indicate significant differences). Q.T.: quantification transition.

Compounds	[M/H]^−^	MS Fragments	Q. T.	Bound Phenolic Compounds			ANOVA
				S-0	S-48	S-72	
Phenolic acids							
1-3-O-dicaffeoylglycerol	415	161, 253, 135	415 → 135	n.d.	n.d.	n.d.	
Caffeic acid	179	135	179 → 135	n.d.	n.d.	n.d.	
Total phenolic acid				n.d.	n.d.	n.d.	
Flavan-3-ols							
Catechin	289	123, 109, 203	289 → 123	n.d.	n.d.	n.d.	
Total flavan-3-ol				n.d.	n.d.	n.d.	
Flavonols							
Taxifolin	303	125, 175, 217	303 → 125	n.d. b	13.02 ± 1.23 a	9.46 ± 1.33 a	*p* < 0.05
Total flavonol				n.d. b	13.02 ± 1.23 a	9.46 ± 1.33 a	*p* < 0.05
Flavones							
Apigenin	269	117, 149, 227	269 → 117	n.d.	n.d.	n.d.	
Hispidulin isomer 1	299	284, 256, 136	299 → 284	n.d. c	17.06 ± 0.72 b	62.32 ± 2.97 a	*p* < 0.05
Hispidulin isomer 2	299	284, 256, 136	299 → 284	7.49 ± 0.17 c	24.34 ± 0.54 b	34.65 ± 1.23 a	*p* < 0.05
Luteolin	285	133, 151, 217	285 → 133	67.65 ± 4.66 c	84.21 ± 0.10 b	103.23 ± 3.65 a	*p* < 0.05
Total flavones				75.14 ± 4.83 c	125.61 ± 1.36 b	200.20 ± 7.85 a	*p* < 0.05
Proanthocyanidins							
Procyanidin dimer	577	289, 407, 425	577 → 289	n.d.	n.d.	n.d.	
Total proanthocyanidins				n.d.	n.d.	n.d.	
Flavanones							
Eriodictyol	287	125, 151, 193	287 → 125	n.d. b	21.64 ± 0.18 a	n.d. b	*p* < 0.05
Naringenin	271	119, 107, 151	271 → 119	11.17 ± 0.15 c	34.41 ± 2.65 b	49.70 ± 1.18 a	*p* < 0.05
Kaempferol	285	199, 175, 217	285 → 199	n.d.	n.d.	n.d.	
Total flavanones				11.17 ± 0.15 b	56.05 ± 2.83 a	49.70 ± 1.18 a	*p* < 0.05
Flavanonols							
Dihydromyricetin 3-O-Rhamnoside	465	285, 275, 303	465 → 285	n.d.	n.d.	n.d.	
Total flavanonols				n.d.	n.d.	n.d.	
3-Deoxyanthocyanidins							
Luteolinidin	269	201, 227, 241	269 → 201	n.d.	n.d.	n.d.	
Apigeninidin	253	209, 117, 181	253 → 209	n.d.	n.d.	n.d.	
5-methoxy-luteolinidin	283	268, 196, 240	283 → 268	n.d.	n.d.	n.d.	
7-methoxy-apigeninidin	267	252, 224, 180	267 → 252	n.d.	n.d.	n.d.	
Total 3-deoxyanthocyanidins				n.d.	n.d.	n.d.	
Total phenol compounds				86.31 ± 4.98 c	194.68 ± 5.42 b	259.36 ± 10.36 a	*p* < 0.05

**Table 5:** Pepsin activity in the absence (w/o extract) or presence of unsprouted (S-0) and sprouted sorghum (48 h, S-48; 72 h, S-72). Pepsin activity is expressed as U/mg enzyme. Data are the means ± SD of two different extractions and triplicate activity measurements. Statistical analysis was conducted by means of one-way ANOVA (*p* < 0.05) with Tukey’s post hoc test (different letters indicate significant differences).

	w/o Extract	S-0	S-48	S-72
Pepsin activity	89.44 ± 4.81 b	115.00 ± 17.40 ab	139.44 ± 18.43 a	148.89 ± 20.09 a

## Data Availability

The data that support the findings of this study are available from the corresponding author upon request.

## Supplementary Materials

The following supporting information can be downloaded at: https://www.mdpi.com/article/10.3390/molecules30173622/s1, Table S1: Soaking, sprouting, and drying parameters.

## Abbreviations

The following abbreviations, without full definitions in the text, are used in this manuscript:

AACC	American Association of Cereal Chemists
HPLC	High-Performance Liquid Chromatography
Da	Dalton
TRIS	Tris(hydroxymethyl)aminomethane

## References

[1] Singh B.K., Delgado-Baquerizo M., Egidi E., Guirado E., Leach J.E., Liu H., Trivedi P. (2023). Climate change impacts on plant pathogens, food security and paths forward. Nat. Rev. Microbiol..

[2] Palavecino P.M., Curti M.I., Bustos M.C., Penci M.C., Ribotta P.D. (2020). Sorghum Pasta and Noodles: Technological and Nutritional Aspects. Plant Foods Hum. Nutr..

[3] Girard A.L., Awika J.M. (2018). Sorghum polyphenols and other bioactive components as functional and health promoting food ingredients. J. Cereal Sci..

[4] Xiong Y., Zhang P., Warner R.D., Fang Z. (2019). Sorghum Grain: From Genotype, Nutrition, and Phenolic Profile to Its Health Benefits and Food Applications. Compr. Rev. Food Sci. Food Saf..

[5] Rashwan A.K., Yones H.A., Karim N., Taha E.M., Chen W. (2021). Potential processing technologies for developing sorghum-based food products: An update and comprehensive review. Trends Food Sci. Technol..

[6] Rodríguez-España M., Figueroa-Hernández C.Y., Figueroa-Cárdenas J.D., Rayas-Duarte P., Hernández-Estrada Z.J. (2022). Effects of germination and lactic acid fermentation on nutritional and rheological properties of sorghum: A graphical review. Curr. Res. Food Sci..

[7] Adebo O.A. (2020). African Sorghum-Based Fermented Foods: Past, Current and Future Prospects. Nutrients.

[8] Khoddami A., Messina V., Vadabalija Venkata K., Farahnaky A., Blanchard C.L., Roberts T.H. (2023). Sorghum in foods: Functionality and potential in innovative products. Crit. Rev. Food Sci. Nutr..

[9] Ari Akin P., Demirkesen I., Bean S.R., Aramouni F., Boyaci I.H. (2022). Sorghum Flour Application in Bread: Technological Challenges and Opportunities. Foods.

[10] Cardone G., Rumler R., Speranza S., Marti A., Schönlechner R. (2021). Sprouting Time Affects Sorghum (*Sorghum bicolor* [L.] Moench) Functionality and Bread-Baking Performance. Foods.

[11] Hugo L.F., Rooney L.W., Taylor J.R.N. (2003). Fermented Sorghum as a Functional Ingredient in Composite Breads. Cereal Chem..

[12] Keyata E.O., Tola Y.B., Bultosa G., Forsido S.F. (2021). Premilling treatments effects on nutritional composition, antinutritional factors, and in vitro mineral bioavailability of the improved Assosa I sorghum variety (*Sorghum bicolor* L.). Food Sci. Nutr..

[13] Elkhalifa A.E.O., Bernhardt R. (2010). Influence of grain germination on functional properties of sorghum flour. Food Chem..

[14] Elkhalifa A.E.O., Bernhardt R. (2018). Combination Effect of Germination and Fermentation on Functional Properties of Sorghum Flour. Curr. J. Appl. Sci. Technol..

[15] Hassan S., Imran M., Ahmad M.H., Khan M.I., Xu C., Khan M.K., Muhammad N. (2020). Phytochemical characterization of ultrasound-processed sorghum sprouts for the use in functional foods. Int. J. Food Prop..

[16] Marchini M., Marti A., Folli C., Prandi B., Ganino T., Conte P., Fadda C., Mattarozzi M., Carini E. (2021). Sprouting of Sorghum (*Sorghum bicolor* [L.] Moench): Effect of Drying Treatment on Protein and Starch Features. Foods.

[17] Marengo M., Bonomi F., Marti A., Pagani M.A., Elkhalifa A.E.O., Iametti S. (2015). Molecular features of fermented and sprouted sorghum flours relate to their suitability as components of enriched gluten-free pasta. LWT—Food Sci. Technol..

[18] Osman M.A. (2004). Changes in sorghum enzyme inhibitors, phytic acid, tannins and in vitro protein digestibility occurring during Khamir (local bread) fermentation. Food Chem..

[19] Saithalavi K.M., Bhasin A., Yaqoob M. (2021). Impact of sprouting on physicochemical and nutritional properties of sorghum: A review. J. Food Meas. Charact..

[20] Li H., Li X., Wang G., Zhang J., Wang G. (2022). Analysis of gene expression in early seed germination of rice: Landscape and genetic regulation. BMC Plant Biol..

[21] Benincasa P., Falcinelli B., Lutts S., Stagnari F., Galieni A. (2019). Sprouted Grains: A Comprehensive Review. Nutrients.

[22] Setia R., Dai Z., Nickerson M.T., Sopiwnyk E., Malcolmson L., Ai Y. (2019). Impacts of short-term germination on the chemical compositions, technological characteristics and nutritional quality of yellow pea and faba bean flours. Food Res. Int..

[23] Li C., Oh S.G., Lee D.H., Baik H.W., Chung H.J. (2017). Effect of germination on the structures and physicochemical properties of starches from brown rice, oat, sorghum, and millet. Int. J. Biol. Macromol..

[24] Teobaldi A.G., Carrillo Parra E.J., Barrera G.N., Ribotta P.D. (2024). The Properties of Damaged Starch Granules: The Relationship Between Granule Structure and Water-Starch Polymer Interactions. Foods.

[25] Salvati D., Paschoalinotto B.H., Mandim F., Ferreira I., Steinmacher N.C., Pereira C., Dias M.I. (2024). Exploring the Impacts of Sorghum (*Sorghum bicolor* L. Moench) Germination on the Flour’s Nutritional, Chemical, Bioactive, and Technological Properties. Foods.

[26] Sharma B., Gujral H.S. (2020). Modifying the dough mixing behavior, protein & starch digestibility and antinutritional profile of minor millets by sprouting. Int. J. Biol. Macromol..

[27] Shah U., Dwivedi D., Hackett M., Al-Salami H., Utikar R.P., Blanchard C., Gani A., Rowles M.R., Johnson S.K. (2021). Physicochemical characterisation of kafirins extracted from sorghum grain and dried distillers grain with solubles related to their biomaterial functionality. Sci. Rep..

[28] Abdelbost L., Morel M.H., Nascimento T.P.D., Cameron L.C., Bonicel J., Larraz M.F.S., Mameri H. (2023). Sorghum grain germination as a route to improve kafirin digestibility: Biochemical and label free proteomics insights. Food Chem..

[29] Egger L., Schlegel P., Baumann C., Stoffers H., Guggisberg D., Brügger C., Dürr D., Stoll P., Vergères G., Portmann R. (2017). Physiological comparability of the harmonized INFOGEST in vitro digestion method to in vivo pig digestion. Food Res. Int..

[30] Di Nunzio M., Loffi C., Montalbano S., Chiarello E., Dellafiora L., Picone G., Antonelli G., Tedeschi T., Buschini A., Capozzi F. (2022). Cleaning the Label of Cured Meat; Effect of the Replacement of Nitrates/Nitrites on Nutrients Bioaccessibility, Peptides Formation, and Cellular Toxicity of In Vitro Digested Salami. Int. J. Mol. Sci..

[31] Kehinde B.A., Majid I., Hussain S. (2022). Isolation of bioactive peptides and multiple nutraceuticals of antidiabetic and antioxidant functionalities through sprouting: Recent advances. J. Food Biochem..

[32] Martinez M., Gómez-Cabellos S., Giménez M.J., Barro F., Diaz I., Diaz-Mendoza M. (2019). Plant Proteases: From Key Enzymes in Germination to Allies for Fighting Human Gluten-Related Disorders. Front. Plant Sci..

[33] Ogbonna A.C., Obi S.K.C., Okolo B.N., Odibo F.J.C. (2004). Purification and some properties of a cysteine proteinase from sorghum malt variety SK5912. J. Sci. Food Agric..

[34] Wang X., Zhao M., Shang P., Liu J., Zhao R. (2024). Effect of Microwave Treatment on Protease Activity, Dough Properties and Protein Quality in Sprouted Wheat. Foods.

[35] Bera I., O’Sullivan M., Flynn D., Shields D.C. (2023). Relationship between Protein Digestibility and the Proteolysis of Legume Proteins during Seed Germination. Molecules.

[36] Hassan S., Imran M., Ahmad N., Khan M.K. (2017). Lipids characterization of ultrasound and microwave processed germinated sorghum. Lipids Health Dis..

[37] Al-Taher F., Nemzer B. (2023). Effect of Germination on Fatty Acid Composition in Cereal Grains. Foods.

[38] Kumar R.R., Bhargava D.V., Pandit K., Goswami S., Mukesh Shankar S., Singh S.P., Rai G.K., Tara Satyavathi C., Praveen S. (2021). Lipase—The fascinating dynamics of enzyme in seed storage and germination—A real challenge to pearl millet. Food Chem..

[39] Wang Y., Sun X., Peng J., Li F., Ali F., Wang Z. (2025). Regulation of seed germination: ROS, epigenetic, and hormonal aspects. J. Adv. Res..

[40] Rai-Kalal P., Tomar R.S., Jajoo A. (2021). H2O2 signaling regulates seed germination in ZnO nanoprimed wheat (*Triticum aestivum* L.) seeds for improving plant performance under drought stress. Environ. Exp. Bot..

[41] Gonçalves J.P., Gasparini K., Picoli E.A.T., Costa M.D.L., Araujo W.L., Zsögön A., Ribeiro D.M. (2024). Metabolic control of seed germination in legumes. J. Plant Physiol..

[42] Amft J., Meissner P.M., Steffen-Heins A., Hasler M., Stöckmann H., Meynier A., Birault L., Velasco J., Vermoesen A., Perez-Portabella I. (2023). Interlaboratory study on lipid oxidation during accelerated storage trials with rapeseed and sunflower oil analyzed by conjugated dienes as primary oxidation products. Eur. J. Lipid Sci. Technol..

[43] Goswami S., Asrani P., Ansheef Ali T.P., Kumar R.D., Vinutha T., Veda K., Kumari S., Sachdev A., Singh S.P., Satyavathi C.T. (2020). Rancidity Matrix: Development of Biochemical Indicators for Analysing the Keeping Quality of Pearl Millet Flour. Food Anal. Methods.

[44] Niro S., D’Agostino A., Fratianni A., Cinquanta L., Panfili G. (2019). Gluten-Free Alternative Grains: Nutritional Evaluation and Bioactive Compounds. Foods.

[45] Guo Y., Li D., Liu T., Liao M., Li Y., Zhang W., Liu Z., Chen M. (2022). Effect of Overexpression of γ-Tocopherol Methyltransferase on α-Tocopherol and Fatty Acid Accumulation and Tolerance to Salt Stress during Seed Germination in *Brassica napus* L.. Int. J. Mol. Sci..

[46] Tarasevičienė Ž., Viršilė A., Danilčenko H., Duchovskis P., Paulauskienė A., Gajewski M. (2019). Effects of germination time on the antioxidant properties of edible seeds. CyTA—J. Food.

[47] Azzi A., Breyer I., Feher M., Pastori M., Ricciarelli R., Spycher S., Staffieri M., Stocker A., Zimmer S., Zingg J.M. (2000). Specific cellular responses to alpha-tocopherol. J. Nutr..

[48] Delgado A., Al-Hamimi S., Ramadan M.F., Wit M.D., Durazzo A., Nyam K.L., Issaoui M. (2020). Contribution of Tocols to Food Sensorial Properties, Stability, and Overall Quality. J. Food Qual..

[49] Nagy R., Kun-Nemes A., Szőllősi E., Bíróné Molnár P., Cziáky Z., Murányi E., Sipos P., Remenyik J. (2024). Physiological potential of different Sorghum bicolor varieties depending on their bioactive characteristics and antioxidant potential as well as different extraction methods. Heliyon.

[50] Bertelli A., Biagi M., Corsini M., Baini G., Cappellucci G., Miraldi E. (2021). Polyphenols: From Theory to Practice. Foods.

[51] Borgonovi S.M., Chiarello E., Pasini F., Picone G., Marzocchi S., Capozzi F., Bordoni A., Barbiroli A., Marti A., Iametti S. (2023). Effect of Sprouting on Biomolecular and Antioxidant Features of Common Buckwheat (*Fagopyrum esculentum*). Foods.

[52] Ti H., Zhang R., Zhang M., Li Q., Wei Z., Zhang Y., Tang X., Deng Y., Liu L., Ma Y. (2014). Dynamic changes in the free and bound phenolic compounds and antioxidant activity of brown rice at different germination stages. Food Chem..

[53] Tomé-Sánchez I., Martín-Diana A.B., Peñas E., Bautista-Expósito S., Frias J., Rico D., González-Maillo L., Martinez-Villaluenga C. (2020). Soluble Phenolic Composition Tailored by Germination Conditions Accompany Antioxidant and Anti-inflammatory Properties of Wheat. Antioxidants.

[54] Liu A.-L., Wang Y.-H., Wang T.-Y., Zhu Y., Wu P., Li L.-J. (2023). Comparative metabolomic profiling of secondary metabolites in different tissues of Euryale ferox and functional characterization of phenylalanine ammonia-lyase. Ind. Crops Prod..

[55] Kaur K., Asthir B. (2022). Regulation of polyphenol catabolism in amelioration of high-temperature stress vis-a-vis antioxidant defense system in wheat. Cereal Res. Commun..

[56] Dicko M.H., Gruppen H., Zouzouho O.C., Traoré A.S., van Berkel W.J.H., Voragen A.G.J. (2006). Effects of germination on the activities of amylases and phenolic enzymes in sorghum varieties grouped according to food end-use properties. J. Sci. Food Agric..

[57] Punia H., Tokas J., Malik A., Bajguz A., El-Sheikh M.A., Ahmad P. (2021). Ascorbate-Glutathione Oxidant Scavengers, Metabolome Analysis and Adaptation Mechanisms of Ion Exclusion in Sorghum under Salt Stress. Int. J. Mol. Sci..

[58] Modgil R., Sood P. (2017). Effect of Roasting and Germination on Carbohydrates and Anti-nutritional Constituents of Indigenous and Exotic Cultivars of Pseudo-cereal (*Chenopodium*). J. Life Sci..

[59] Santos S.C., Fortes G.A.C., Camargo L.T.F.M., Camargo A.J., Ferri P.H. (2021). Antioxidant effects of polyphenolic compounds and structure-activity relationship predicted by multivariate regression tree. LWT.

[60] Platzer M., Kiese S., Tybussek T., Herfellner T., Schneider F., Schweiggert-Weisz U., Eisner P. (2022). Radical Scavenging Mechanisms of Phenolic Compounds: A Quantitative Structure-Property Relationship (QSPR) Study. Front. Nutr..

[61] Feizollahi E., Mirmahdi R.S., Zoghi A., Zijlstra R.T., Roopesh M.S., Vasanthan T. (2021). Review of the beneficial and anti-nutritional qualities of phytic acid, and procedures for removing it from food products. Food Res. Int..

[62] Brouns F. (2021). Phytic Acid and Whole Grains for Health Controversy. Nutrients.

[63] Elliott H., Woods P., Green B.D., Nugent A.P. (2022). Can sprouting reduce phytate and improve the nutritional composition and nutrient bioaccessibility in cereals and legumes?. Nutr. Bull..

[64] Maldonado-Alvarado P., Pavón-Vargas D.J., Abarca-Robles J., Valencia-Chamorro S., Haros C.M. (2023). Effect of Germination on the Nutritional Properties, Phytic Acid Content, and Phytase Activity of Quinoa (*Chenopodium quinoa* Willd). Foods.

[65] Silva V.M., Putti F.F., White P.J., Reis A.R.D. (2021). Phytic acid accumulation in plants: Biosynthesis pathway regulation and role in human diet. Plant Physiol. Biochem..

[66] Kårlund A., Paukkonen I., Gómez-Gallego C., Kolehmainen M. (2021). Intestinal Exposure to Food-Derived Protease Inhibitors: Digestion Physiology- and Gut Health-Related Effects. Healthcare.

[67] Stanforth K., Wilcox M., Chater P., Brownlee I., Zakhour M., Banecki K., Pearson J. (2021). Pepsin properties, structure, and its accurate measurement: A narrative review. Ann. Esophagus.

[68] Gomez-Urios C., Siroli L., Grassi S., Patrignani F., Blesa J., Lanciotti R., Frígola A., Iametti S., Esteve M.J., Di Nunzio M. (2025). Sustainable valorization of citrus by-products: Natural deep eutectic solvents for bioactive extraction and biological applications of Citrus sinensis peel. Eur. Food Res. Technol..

[69] Borgonovi S.M., Perugino F., Dellafiora L., Annunziata F., Pedroni L., Galaverna G., Pinto A., Dallavalle S., Iametti S., Di Nunzio M. (2025). Assessing the impact of food-derived bioactives on digestive proteases by in vitro and in silico approaches. Food Funct..

[70] Budhwar S., Sethi K., Chakraborty M. (2020). Efficacy of germination and probiotic fermentation on underutilized cereal and millet grains. Food Prod. Process. Nutr..

[71] Dia V.P., Gomez T., Vernaza G., Berhow M., Chang Y.K., de Mejia E.G. (2012). Bowman-Birk and Kunitz protease inhibitors among antinutrients and bioactives modified by germination and hydrolysis in Brazilian soybean cultivar BRS 133. J. Agric. Food Chem..

[72] Wilson K.A. (1988). The proteolysis of trypsin inhibitors in legume seeds. Crit. Rev. Biotechnol..

[73] Singh A., Bobade H., Sharma S., Singh B., Gupta A. (2021). Enhancement of Digestibility of Nutrients (In vitro), Antioxidant Potential and Functional Attributes of Wheat Flour Through Grain Germination. Plant Foods Hum. Nutr..

[74] Azeez S.O., Chinma C.E., Bassey S.O., Eze U.R., Makinde A.F., Sakariyah A.A., Okubanjo S.S., Danbaba N., Adebo O.A. (2022). Impact of germination alone or in combination with solid-state fermentation on the physicochemical, antioxidant, in vitro digestibility, functional and thermal properties of brown finger millet flours. LWT.

[75] Yang F., Basu T.K., Ooraikul B. (2001). Studies on germination conditions and antioxidant contents of wheat grain. Int. J. Food Sci. Nutr..

[76] Setia R., Dai Z., Nickerson M.T., Sopiwnyk E., Malcolmson L., Ai Y. (2020). Properties and bread-baking performance of wheat flour composited with germinated pulse flours. Cereal Chem..

[77] Sharanagat V.S., Nema P.K. (2023). Bread preparation by partial replacement of wheat by germinated sorghum. Food Sci. Technol. Int..

[78] Nkurikiye E., Xiao R., Tilley M., Siliveru K., Li Y. (2023). Bread-making properties of different pulse flours in composites with refined wheat flour. J. Texture Stud..

[79] Tagliasco M., Font G., Renzetti S., Capuano E., Pellegrini N. (2024). Role of particle size in modulating starch digestibility and textural properties in a rye bread model system. Food Res. Int..

[80] Soglia F., Mazzoni M., Zappaterra M., Di Nunzio M., Babini E., Bordini M., Sirri F., Clavenzani P., Davoli R., Petracci M. (2019). Distribution and Expression of Vimentin and Desmin in Broiler Pectoralis major Affected by the Growth-Related Muscular Abnormalities. Front. Physiol..

[81] Di Nunzio M., Betoret E., Taccari A., Dalla Rosa M., Bordoni A. (2020). Impact of processing on the nutritional and functional value of mandarin juice. J. Sci. Food Agric..

[82] Di Nunzio M., Loffi C., Chiarello E., Dellafiora L., Picone G., Antonelli G., Di Gregorio C., Capozzi F., Tedeschi T., Galaverna G. (2022). Impact of a Shorter Brine Soaking Time on Nutrient Bioaccessibility and Peptide Formation in 30-Months-Ripened Parmigiano Reggiano Cheese. Molecules.

[83] Saini R.K., Prasad P., Shang X., Keum Y.S. (2021). Advances in Lipid Extraction Methods-A Review. Int. J. Mol. Sci..

[84] Ghini V., Di Nunzio M., Tenori L., Valli V., Danesi F., Capozzi F., Luchinat C., Bordoni A. (2017). Evidence of a DHA Signature in the Lipidome and Metabolome of Human Hepatocytes. Int. J. Mol. Sci..

[85] Bub A., Malpuech-Brugère C., Orfila C., Amat J., Arianna A., Blot A., Di Nunzio M., Holmes M., Kertész Z., Marshall L. (2019). A Dietary Intervention of Bioactive Enriched Foods Aimed at Adults at Risk of Metabolic Syndrome: Protocol and Results from PATHWAY-27 Pilot Study. Nutrients.

[86] Di Nunzio M., Valli V., Bordoni A. (2011). Pro- and anti-oxidant effects of polyunsaturated fatty acid supplementation in HepG2 cells. Prostaglandins Leukot. Essent. Fat. Acids.

[87] Abeyrathne E., Nam K., Ahn D.U. (2021). Analytical Methods for Lipid Oxidation and Antioxidant Capacity in Food Systems. Antioxidants.

[88] Verma A., Singh S., Thawait L.K., Mahatma M.K., Singh A.L. (2022). An expedient ion chromatography based method for high-throughput analysis of phytic acid in groundnut kernels. J. Food Sci. Technol..

[89] Urbinati E., Di Nunzio M., Picone G., Chiarello E., Bordoni A., Capozzi F. (2021). The Effect of Balsamic Vinegar Dressing on Protein and Carbohydrate Digestibility is Dependent on the Food Matrix. Foods.

[90] Di Nunzio M., Picone G., Pasini F., Chiarello E., Caboni M.F., Capozzi F., Gianotti A., Bordoni A. (2020). Olive oil by-product as functional ingredient in bakery products. Influence of processing and evaluation of biological effects. Food Res. Int..

