# Supplementary material for: Exploring Germination to Unlock the Nutritional Potential of Sorghum (Sorghum bicolor)
Source: Molecules. 2025 Sep 4;30(17):3622. doi: 10.3390/molecules30173622 (PMC12430075; doi:10.3390/molecules30173622)
Supplement: Supplementary file 1 [file molecules-30-03622-s001.zip › molecules-3787564-supplementary.pdf]

**Sample**			**Soaking**				**Sprouting**				**Drying**	
Name	Seed quantity (kg)		Seed:water ratio (w:w)	Time (h)	Temp. (°C)		Time (h)	Temp. (°C)	Relative humidity (%)		Time (h)	Temp. (°C)
S-0	2.5		1:3	16	27		0	27	90		8	50
S-48	2.5		1:3	16	27		48	27	90		8	50
S-72	2.5		1:3	16	27		72	27	90		8	50

Supplementary table S1. Soaking, sprouting, and drying parameters
